# The predictive role of histopathological findings in renal insufficiency and complete remission in a sample of Iranian adults with primary focal segmental glomerulosclerosis

**Published:** 2010

**Authors:** Diana Taheri, Ali Chehrei, Pargol Samanianpour, Shohreh Sadrarhami, Ammar Hassanzadeh Keshteli, Shahrzad Shahidi

**Affiliations:** a Department of Pathology, School of Medicine, Isfahan University of Medical Sciences, Isfahan, Iran; b School of Medicine, Arak University of Medical Sciences, Arak, Iran; c Medical Students Research Committee, School of Medicine, Isfahan University of Medical Sciences, Isfahan, Iran; d Vice Chancellery for Research, Isfahan University of Medical Sciences, Isfahan, Iran; e Division of Nephrology, Department of Internal Medicine, Isfahan University of Medical Sciences, Isfahan, Iran

**Keywords:** Focal Segmental Glomerulosclerosis, Mesangial Hypercellularity, Hyalinosis, Global Scar, Renal Insufficiency, Remission

## Abstract

**BACKGROUND:**

Primary focal segmental glomerulosclerosis (FSGS) is defined by the presence of proteinuria, often in nephrotic range and pathologically by segmental scars (SS). The aim of this study is to identify the possible predictors of complete remission or progression to chronic kidney disease in Iranian adults with primary focal segmental glomerulosclerosis.

**METHODS:**

In this historical cohort study, pathological findings of 50 patients with primary FSGS were reviewed by single renal pathologist without knowing about patients’ identities or outcomes. Patients were divided based on their histopathological findings and outcomes were compared among these groups.

**RESULTS:**

There were significant differences in the complete remission rate in subjects with and without mesangial hypercellularity (p < 0.05), and in patients with and without hyalinosis (p < 0.05). According to the cut off points based on ROC curve analysis for the quantitative data, there was significant difference in renal insufficiency between the patients with and without global scars more than 12% (p < 0.05). Also multiple logistic regression analysis strongly suggests the association of mesangial hypercellularity and global scar with no complete remission and progression to renal insufficiency, respectively.

**CONCLUSIONS:**

In the studied patients, presence of mesangial hypercellularity and hyalinosis has been suggested as prognostic factors for lower remission rate. According to multivariate analysis, only mesangial hypercellularity and global scar were found to act as independent prognostic predictors of lower complete remission rate and progression to renal insufficiency in patients with FSGS, respectively.

Primary focal segmental glomerulosclerosis (FSGS) is defined by the presence of proteinuria, often in nephrotic range and pathologically by segmental scars (SS) involving some but not all glomeruli. FSGS is distinguished from minimal change disease by hematuria, hypertension and renal insufficiency (RI) at presentation, poor response to corticosteroid therapy, and a progressive course to end-stage renal disease (ESRD).^1^ There is evidence that FSGS is becoming more common. Several publications suggest that the prevalence of idiopathic FSGS in adults may be increasing from 2.5-4% of renal biopsies in 1970 to 12.2-18.7% in the present decade, making FSGS the most common diagnosis based on native kidney biopsies and confirming its increasing importance as a cause of end-stage renal disease.^2^,^3^

The classic pathologic description of FSGS is a pattern of injury defined by a SS which involves some but not all glomeruli. In a better form, FSGS is described as segmental scarring involving less than 50% of the glomerulus and affecting less than 50% of glomeruli. Even one glomerulus is sufficient for diagnosis. When all of the secondary causes of this pattern of injury are excluded, the remaining patients receive a diagnosis of primary FSGS.^4^ In addition to glomerular scars, several other morphologic features such as global scars (GS), presence of foam cells, glomerular hyalinization and mesangial hypercellularity (> 3 cells per mesangial area) have been described in FSGS.

There are some clinical prognostic criteria such as serum creatinine (Cr) level and nephrotic syndrome at the time of biopsy.^5^,^6^ However, the prognostic and therapeutic utility of microscopic features remains controversial,^7^,^8^ largely because studies that have assessed the clinical relevance of the histologic variants of primary FSGS in nephrotic patients are few and conflicting.^6^,^9^–^14^

In this article, the results of a historical cohort study is reported which was designed to identify the histological findings that are possible predictors of remission or progression to RI in Iranian adults with primary FSGS.

## Methods

This historical cohort study was conducted during 2004 to 2008 in Department of Pathology of Al-Zahra Hospital, Isfahan University of Medical Sciences (Iran). All kidney biopsies were taken using 16 or 18 gauge needles. In each case, glass slides stained with hematoxylineosin, Masson trichrome, periodic acid-Schiff (PAS) and methenamine silver periodic acid-Schiff (Jones) were available for review. Light microscopy findings were analyzed by a renal pathologist without knowledge about patients’ identities or outcomes.

The diagnosis of primary FSGS was made according to the following criteria: (I) a lesion involving some of the glomeruli in the biopsy with others remaining uninvolved, (II) the involved glomeruli having a portion that has undergone collapse of capillaries with obliteration of capillary lumina with or without adhesions, and (III) no clinical or pathological evidence of primary disease that might produce secondary FSGS.^14^ Only patients with FSGS as their initial glomerular lesion were included in the study. The medical records were reviewed, and patients with evidence of systemic disease, other diseases associated with primary or secondary glomerulopathy, or a history of reflux nephropathy, nephrectomy, solitary kidney, human immunodeficiency virus infection or intravenous drug abuse were excluded. All of the cases had BMI < 30 which made them eligible to be included in the study.

The following features were recorded: the proportion of glomeruli with SS, the proportion of glomeruli with GS, the proportion of glomeruli with hyalinosis, the location of each SS in relation to the vascular and tubular poles of the tuft, the presence of foam cells, the presence of mesangial hypercellularity (> 3 cells per mesangial area) and sclerosis, the presence of epithelial cell proliferation (visceral and parietal), the presence of synechiae with the Bowman’s capsule, the presence of interstitial fibrosis, and the presence of tubular atrophy.

Laboratory data of all patients with “classic” FSGS on renal biopsy were reviewed and obtained throughout their follow-up. The data included serum Cr, level of proteinuria, and date of need for hemodialysis or kidney transplant.

The following definitions were used for classification of the patients: normal plasma Cr as Cr ≤ 1.3 mg/dl; RI as Cr > 1.3 mg/dl; and end stage renal disease as need for hemodialysis or renal replacement therapy.^10^

Complete remission (CR) was defined as a urine protein of ≤ 0.25 g/24h and no complete remission was considered when proteinuria was more than 0.25 g/24h (including subjects with partial remission and resistant cases).^15^

In this study, patients were divided according to the histological findings and outcomes were compared (RI and complete remission) between them.

All data were analyzed by SPSS version 15 (SPSS Inc, Chicago IL, USA). Simple descriptive techniques were used to describe variables among the participants. The K-S and Levene’s tests were applied to verify normal distribution and the quality of variances. According to the results of the tests, student t test or Mann-Whitney U test were used to compare the quantitative data in grouping variables. Chi square test was used to find the relationship between qualitative data. Receiver Operating Characteristic (ROC) curves were used to determine the optimal cut-off values for quantitative risk factors. Logistic regression analysis was used for multivariate analysis.

All the procedures were performed in accordance with the Helsinki Declaration (1964, amended in 1975 and 1983).

## Results

On reviewing the renal biopsies and according to the exclusion criteria, the diagnosis of primary FSGS was confirmed in 50 patients, who were included in the study.

There were 31 men (62%) and 19 women (38%). The mean (± 2 SE) of age irrespective of sex was 33.28 (29.18-37.38) years. The mean (± 2 SE) of age was 32.49 (27.03-37.95) and 27.24 (27.22-27.26) years in men and women, respectively.

All the histopathological findings of primary FSGS were evaluated. The overall average number of glomeruli for evaluation by light microscopy was 17.88 (15.28-20.48). The means of the percents of glomeruli with SS and GS were 29.2 % (22.6-35.8%) and 13.8 % (9.27-16.87%), respectively.

The mean duration of follow-up was 35.96 (27.36-44.56) months. The duration of follow-up was similar among different groups. At the end of the follow-up one patient died and none of the patients progressed to ESRD.

Incidence of complete remission was 15 (30%) and no complete remission was 35 (70%). Also 4 patients (8%) persisted nephrotic-rang proteinuria and 17 patients (34%) progressed to RI (Cr > 1.3).

The percentage of RI and complete remission in patients with and without each of the pathological findings are shown in table 1.

**Table 1:** The percentage of renal insufficiency and complete remission in primary FSGS patients with and without the histopathological findings

Histopathological findings	Status	Renal insufficiency				Complete remission			
		Yes	No	P value	Risk ratio	Yes	No	P value	Risk ratio*
Interstitial fibrosis	Yes	21 (56.8%)	16 (43.2%)	0.33	0.63	10(25.6%)	29(74.4%)	0.52	1.36
	No	9 (90%)	1 (10%)			5 (45.5%)	6 (54.5%)		

Synechiae with adhesion to Bowman’s capsule	Yes	10 (55.6%)	8 (44.4%)	0.60	0.81	5 (26.3%)	14 (73.7%)	0.80	1.09
	No	20 (69%)	9 (31%)			10 (32.3%)	21 (67.7%)		

Mesangial hypercellularity	Yes	15 (62.5%)	9 (37.5%)	0.83	0.96	5 (18.5%)	22 (81.5%)	0.04	1.87
	No	15 (65.2%)	8 (34.8%)			10 (43.5%)	13 (56.5%)		

Mesangial sclerosis	Yes	12 (57.1%)	9 (42.9%)	0.06	0.83	5 (22.7%)	17 (77.3%)	0.08	1.2
	No	18 (69.2%)	8 (30.8%)			10 (35.7%)	18 (64.3%)		

Tubular atrophy	Yes	21 (58.3%)	15 (41.7%)	0.12	0.71	11 (28.9%)	27 (71.1%)	0.19	1.07
	No	9 (81.8%)	2 (18.2%)			4 (33.3%)	8 (66.7%)		

Presence of glomerular hyalinosis	Yes	8 (72.7%)	3 (27.3%)	0.07	1.19	1 (9.1%)	10 (90.9%)	0.05	1.42
	No	22 (61.1%)	14 (38.9%)			14 (35.9%)	25 (64.1%)		

Presence of global scars ≥ 12%	Yes	13 (59.1%)	9 (40.9%)	0.02	3.69	4 (18.2%)	18 (81.8%)	0.46	1.35
	No	4 (16%)	21 (84%)			11 (39.3%)	17 (60.7%)		

* For no complete remission (including subjects with partial remission and resistant cases)

The frequencies of complete remission in groups with and without mesangial hyperplasia were 5 (18.5%) and 10 (43.5%) and in groups with and without hyalinosis were 1 (9%) and 14 (35.9%). The rate of complete remission was significantly different between the groups with and without mesangial hyperplasia (p < 0.05), and between the cases with and without hyalinosis (p < 0.05).

There was no significant difference in the outcomes (i.e. RI and CR) between the groups with respect to other histological findings.

### 

#### ROC Curve Analysis

For the analysis of the quantitative histological findings (including percent of glomeruli with GS and SS), ROC curve analysis was used to determine the best cut-off points of these variables as predictive factors of RI and CR in primary FSGS.

For predicting “no complete remission”, the area under curve (AUC) of ROC curve of GS and SS were 0.59 and 0.62, respectively. So the cut-off point of 0.20 (with sensitivity of 63% and specificity of 60%) for SS and the cut off points of 0.10 (with sensitivity of 58% and specificity of 64%) for GS was accepted.

Also to predict RI, the AUC was 0.48 for SS and 0.79 for GS. So the best cut-off point for GS to predict RI was 12% (with sensitivity and specificity of 70%).

Then patients were divided according to these cut-off points. The frequency of RI in the group with GS ≤ 12% was 13 (59.1%) and the frequency of RI in the group with GS ≤ 12% was 4 (16%). So there was significant difference in RI between the patients with and without GS more than 12% (p < 0.05). Also the frequency of CR in group with GS ≥ 12% was 4 (18.2%) and the frequency of CR in group with the GS ≤ 12% was 11 (39.3%). Therefore, there was no significant difference in CR between the patients with and without the presence of GS more than 12% (p > 0.05).

In multiple logistic regression analysis, to determine the predictive variables for the “no complete remission”, mesangial hyperplasia was the only variable which entered the model (β = 1.74), and to determine the predictive variables for the RI, GS was the only variable entering the model (β = 8.65). No significant relationship has been found between patients’ histopathological findings and their GFR.

## Discussion

Patients with FSGS are considered to have a poor prognosis.^14^ However, FSGS is not a single disease entity and different outcomes have been seen in patients. An accurate estimate of the prognosis of chronic diseases such as FSGS is particularly important for several reasons. First, prognostic information is useful to advise patients of the probable outcome of disease. Second, knowledge about the prognosis for an individual patient or subgroups of patients can be used as a guide for selecting therapeutic options. Third, identification of the variables with most influence on the rate of progression to chronic kidney disease (CKD) may lead to important etiologic and pathologic insights and allow the characterization of relevant therapeutic targets.^15^ Progression of FSGS to RI depends on various clinical and laboratory factors that have been taken into account in previous studies. Few studies have investigated the prognostic significance of morphologic factors.^14^

In this study, different histopathological findings in Iranian patients with FSGS were investigated to find out some prognostic factors for outcome (RI and complete remission) in these patients.

In the present series, there was a significant association between the presence of mesangial hypercellularity and lower complete remission rate. In agreement with these findings, mesangial hypercellularity has been proposed to indicate a subtype of primary minimal change disease (MCD) with poorer prognosis in other studies.^16^ Cho et al defined the risk of progressive renal disease based on the presence of mesangial hypercellularity, thus directing vigilant follow up and more aggressive treatment.^17^ In contrast to this statement, Alexapoulos et al showed that active glomerular lesions such as mesangial epithelial cell hyperplasia were more often observed in responders to treatment.^18^ According to Ostalska-Nowicka et al, lack of unequivocal criteria for the morphologic definition of mesangial hypercellularity makes it difficult for one to estimate its significance in short or long term prognosis. Moreover, children with FSGS and mesangial hypercellularity do not show a worse prognosis than those with typical FSGS. Thus, diffuse mesangial proliferations do not appear to impart specific prognosis significance in either MCD or FSGS, or to differentiate between apparent MCD and FSGS.^16^

In the present study, no significant association has been found between the presence of interstitial fibrosis and rate of RI in FSGS cases. This finding is against those of other studies.^19^ According to the findings of Glick study, the presence of interstitial fibrosis is the only significant, positive predictor of progression to kidney failure among the histopathological features present on biopsy.^20^

Present findings suggest that the presence of glomeruli with SS more than 20% and GS more than 12% is associated with lower rate of remission. In addition, presence of SS and GS has led to RI in this series. In contrast with this result, Korbet et al state neither SS nor GS as predictors of outcome in FSGS group.^19^ This statement has also been confirmed in other studies.^21^

In the present study, complete remission rate has been lower in the subgroup of cases with hyalinosis. Dumoulin et al concurred that hyalinosis should be considered a feature of FSGS with distinctly poor prognosis,^21^ while other studies don’t agree with this finding.^19^

None of the other histological features evaluated in this study were independently significant as predictors of RI and remission rates, which is along the lines of findings of previous researches.^22^

While age at disease onset and sex were associated with FSGS prognosis in several studies,^2^,^21^ present results showed no significant difference in RI and remission rates with respect to age and gender.

Multivariate analysis strongly suggested that presence of mesangial hypercellularity and GS were independent prognostic factors for RI and no complete remission in primary FSGS. However, a full assessment of the relative risk will require larger cohorts of patients and longer follow-up duration which were limitations for this study.

## Conclusions

In this study, the presence of mesangial hypercellularity and hyalinosis were prognostic factors for lower remission rate. According to multivariate analysis, only the presence of mesangial hypercellularity and GS are suggested as independent prognostic predictors to lower complete remission rate and progression to RI in patients with FSGS, respectively.
